# Birth characteristics and risk of Ewing sarcoma

**DOI:** 10.1007/s10552-023-01737-4

**Published:** 2023-06-19

**Authors:** Joseph L. Wiemels, Rong Wang, Qianxi Feng, Amy C. Yee, Libby M. Morimoto, Catherine Metayer, Xiaomei Ma

**Affiliations:** 1 Center for Genetic Epidemiology, Norris Comprehensive Cancer Center, University of Southern California, Keck School of Medicine, Norris Research Tower (NRT) 1506A, 1450 Biggy St, Los Angeles, CA 90033 USA; 2 Department of Chronic Disease Epidemiology, Yale School of Public Health, New Haven, CT USA; 3 Department of Epidemiology, School of Public Health, University of California Berkeley, Berkeley, CA USA

**Keywords:** Ewing sarcoma, Childhood cancer, Epidemiology, Birth characteristics, Birthweight

## Abstract

**Purpose:**

The incidence of Ewing sarcoma varies according to race and ethnicity, and genetic susceptibility is known to affect disease risk. Apart from these factors, the etiology of Ewing sarcoma is largely unknown.

**Methods:**

We compared the birth characteristics of a population-based series of 556 Ewing sarcoma cases born in California in 1978–2015 and diagnosed in 1988–2015 with those of 27,800 controls selected from statewide birth records and frequency-matched to cases on the year of birth, using multivariable logistic regression models. We also assessed whether Ewing sarcoma clustered within families.

**Results:**

Compared to non-Hispanic White subjects, Black (odds ratio [OR] = 0.07, 95% confidence interval [CI] 0.03–0.18), Asian (OR = 0.57, 95% CI 0.41–0.80), and Hispanic (OR = 0.73, 95% CI 0.62–0.88) individuals had a significantly lower risk of Ewing sarcoma. Race and ethnicity differences were more profound for metastatic Ewing sarcoma. Birthweight was also identified as a significant risk factor (OR = 1.09, 95% CI 1.00–1.18 for each 500 g increase in birthweight). A separate family-based cancer clustering analysis did not suggest any strong role for familial predisposition alleles.

**Conclusions:**

This population-based study with minimal selection bias provides support for a role of accelerated fetal growth in the etiology of Ewing sarcoma in addition to more precise estimates of racial and ethnic variations in disease risk. This comparatively large analysis of birth characteristics and Ewing sarcoma in a multiethnic population should stimulate further investigations into genetic and environmental causes.

**Supplementary Information:**

The online version contains supplementary material available at 10.1007/s10552-023-01737-4.

## Introduction

Ewing sarcoma is a rare, high grade, osteolytic bone tumor, second to osteosarcoma in incidence among malignant bone tumors that present in children and young adults. The disease is often diagnosed in the second decade of life and is rare in children younger than 5 years or in adults over the age of 30 [1]. Ewing sarcoma is curable in the majority of patients by a combination of surgery, radiation, neoadjuvant and adjuvant chemotherapy, but long-term sequelae such as musculoskeletal abnormalities and cardiac outcomes can result from treatment or the disease itself [2].

Ewing sarcoma has several well-known epidemiologic characteristics. Most notably, it is extremely rare in individuals of African descent and also has far lower incidence in Asian than in non-Hispanic White individuals [3]. Hispanic individuals display an intermediate incidence between non-Hispanic White and Black persons [3]. In contrast to osteosarcoma, the risk of Ewing sarcoma was not associated with higher birthweight in a meta-analysis of prior epidemiology studies [4].

In this study, we examined the relationship of Ewing sarcoma to a variety of birth characteristics, using registry resources from California, which is the most populous state in the United States and is very diverse in terms of race and ethnicity. This analysis benefits from a population-based design, a relatively large sample size for a rare malignancy, and a very low likelihood of selection bias, providing precise estimates of racial and ethnic variations in incidence while accounting for a broad spectrum of birth characteristics.

## Methods

We constructed two separate datasets by merging data from the California Cancer Registry (CCR) and California vital statistics (birth) records to examine birth characteristics and familial cancer clustering, respectively.

### Case–control analysis

We identified a total of 556 Ewing sarcoma cases who were born in California during 1978–2015, diagnosed with Ewing sarcoma (International Classification of Diseases for Oncology, 3rd edition, ICD-O-3 code: 9260) at the age of 0–35 years during 1988–2015, and reported to the CCR. Statewide birth records maintained by the California Department of Public Health were used to randomly select 50 times as many control subjects (n = 27,800) who are frequency-matched to the cases by year of birth; none of the controls had been diagnosed with any type of cancer up to the age of 35 years based on CCR records.

For all cases and controls, data on the following variables were retrieved from their birth records: sex, race and ethnicity (categorized as non-Hispanic White, non-Hispanic Black, Hispanic/Latino, non-Hispanic Asian/Pacific Islander, other), birthweight, gestational age, birth plurality, birth order, mode of delivery (vaginal or cesarean), year of birth, maternal age, maternal education, mother’s place of birth (United States or foreign), maternal history of miscarriage or stillbirth (yes/no), maternal complication during pregnancy (yes/no), and maternal history of cesarean delivery (yes/no). A multivariable unconditional logistic regression analysis was performed with case status as the outcome and all birth characteristics described above as independent variables. Incorporation of variables one by one did not alter odds ratios by more than 10%, suggesting a low likelihood of potential confounding. In addition, stratified analyses were performed for larger racial and ethnic groups (Hispanic and non-Hispanic White) as well as by age at diagnosis, histology subtype, and localized or regional disease (CCR variable SUMSTAGE = 1–4) versus metastatic disease (SUMSTAGE = 7). Race and ethnicity were first delineated as Hispanic/non-Hispanic, and secondarily by race, using two separate data fields from birth records. Therefore, “Hispanic subjects” may include individuals of any race, and “non-Hispanic subjects” include persons of White, Black, Asian/Pacific Islander, or other race.

### Family-based analysis

To assess potential familial aggregation that may reflect genetic predisposition to cancer, we ascertained the siblings of younger Ewing sarcoma patients (aged 0–19 years at diagnosis, 353 cases) from the statewide birth records in 1978–2015 and examined whether any of them had been diagnosed with any type of cancer per CCR record. We calculated standardized incidence ratios (SIR) as previously described [5] for siblings’ relative risk by dividing the observed number of cancer cases by the expected number of cases among siblings based on age-specific cancer incidence rates derived from the Surveillance, Epidemiology and End Results (SEER) program [6]. Similar analyses were performed for comparison on additional sarcomas including osteosarcoma (ICD-O-3 codes: 9180-83, 9185-87, and 9192-95, 576 cases), rhabdomyosarcoma (ICD-O-3 codes: 8900-02, 8910, 8912, 8920, 8991, 719 cases), and synovial sarcoma (ICD-O-3 codes: 9040-9043, 127 cases).

All tests were two-sided with an alpha of 0.05 and were conducted using SAS Version 9.4 (SAS Inc. Cary, North Carolina).

## Results

Of the 556 Ewing sarcoma cases identified, 435 were classified as bone tumors and 121 as soft tissue sarcomas. One hundred eighty-two patients exhibited distant metastatic disease at diagnosis, and 352 had localized or regional disease (of which most were in situ, with 16 having presence of tumor in local lymph nodes). Bone tumors were most often located in long bones (42%) or the pelvic, sacrum or coccyx bones (26%). Soft tissue Ewing sarcomas were most often located in the thorax (24%) or lower limb and hip (22%). Ewing sarcoma was 1.3 times more common in males than in females (Table 1). When comparing the incidence of Ewing sarcoma across racial and ethnic groups, the most notable observation is the known deficit of cases among Black individuals: only four cases (0.7% of the total, Table 1) whereas population controls matched on birth year included 8.1% Black subjects (odds ratio [OR] = 0.07, 95% confidence interval [CI] 0.03–0.18 when comparing Black to non-Hispanic White subjects). A reduced risk was also observed for Hispanic and Asian study subjects compared to non-Hispanic White subjects (OR = 0.73, 95% CI 0.62–0.88, and OR = 0.57, 95% CI 0.41–0.80, respectively). A multivariable analysis which included additional birth characteristics attenuated the ORs slightly in Hispanic and Asian but not Black study subjects (Table 2). We note that our grouping of Hispanic individuals included all races, and the lower risks may be related to lower African ancestry among cases; however, Hispanic individuals included only a small percentage of Black persons (0.84% cases and 1.62% controls). Data on the race and ethnicity of parents were available from birth records, and all four Black Ewing sarcoma cases had both parents who were Black. Fourteen percent of Black controls had one parent of a different race or ethnicity, and similar rates of discordant parentage were observed among Asian and Hispanic individuals. We did not attempt further sub-grouping of racial and ethnic groups given the small numbers of subjects with discordant parentage and the lack of available genetic information to inform ancestral evaluation.

**Table 1:** Characteristics of Ewing sarcoma cases and controls, California, 1978–2015

	Case		Control		p*
	*N*	%	*N*	%	
Overall	556		27,800		
Sex					
Female	232	41.7	13,396	48.2	<.01
Male	324	58.3	14,404	51.8	
Race and ethnicity					
Non-Hispanic White	267	48.0	10,141	36.5	<.01
Non-Hispanic Black	4	0.7	2,238	8.1	
Hispanic	238	42.8	12,310	44.3	
Non-Hispanic Asian	41	7.4	2,713	9.8	
Other	6	1.1	398	1.4	
Birth weight (grams)					
250–2499	25	4.5	1,652	5.9	0.02
2500–2999	69	12.4	4,289	15.4	
3000–3499	202	36.3	10,538	37.9	
3500–3999	182	32.7	8,281	29.8	
≥ 4000	78	14.0	3,040	10.9	
Gestational age (weeks)					
22–36	42	7.6	2,485	8.9	0.70
37–41	421	75.7	20,733	74.6	
42–44	56	10.1	2,667	9.6	
Unknown	37	6.7	1,915	6.9	
Birth plurality					
Singleton	541	97.3	27,145	97.6	0.60
Multiple	15	2.7	655	2.4	
Birth order					
1^st^	211	37.9	11,186	40.2	0.09
2^nd^	200	36.0	8,783	31.6	
3rd and higher	145	26.1	7,831	28.2	
Mode of delivery					
Vaginal	434	78.1	21,727	78.2	0.96
Cesarean	122	21.9	6,073	21.8	
Year of birth					
1978–1982	91	16.4	4,550	16.4	1.00
1983–1987	111	20.0	5,550	20.0	
1988–1992	122	21.9	6,100	21.9	
1993–2014	232	41.7	11,600	41.7	
Maternal age (years)					
< 20	56	10.1	3,194	11.5	0.29
20–24	134	24.1	7,385	26.6	
25–29	166	29.9	8,040	28.9	
30–34	139	25.0	6,054	21.8	
≥ 35	61	11.0	3,127	11.2	
Maternal education (years)					
< 9	41	7.4	2,381	8.6	0.08
9–11	68	12.2	3,155	11.3	
12	87	15.6	5,295	19.0	
13–15	81	14.6	3,384	12.2	
≥ 16	72	12.9	3,185	11.5	
Unknown	207	37.2	10,400	37.4	
Mother's place of birth					
United States	358	64.4	16,705	60.1	0.04
Foreign	198	35.6	11,095	39.9	
Maternal history of miscarriage/stillbirth					
No	459	82.6	23,027	82.8	0.80
Yes	97	17.4	4,731	17.0	
Unknown	0		42	0.2	
Maternal complication during pregnancy					
No	455	81.8	23,069	83.0	0.44
Yes	90	16.2	4,172	15.0	
Unknown	11	2.0	559	2.0	
Maternal history of cesarean delivery					
No	506	91.0	25,022	90.0	0.43
Yes	41	7.4	2,310	8.3	
Unknown	9	1.6	468	1.7	
Paternal age (years)					
< 25	112	20.1	6,534	23.5	0.08
25–29	139	25.0	7,403	26.6	
30–34	156	28.1	6,554	23.6	
35–39	80	14.4	3,731	13.4	
≥ 40	40	7.2	2,114	7.6	
Unknown	29	5.2	1,464	5.3	

*p values were derived from chi-square tests

**Table 2:** Odds ratios for Ewing sarcoma in relation to birth characteristics, California, 1978–2015

	Case	Control	Unadjusted			Adjusted^a^		
	*n* (%)	*n* (%)	OR	95% CI	*p*	OR	95% CI	*p*
Overall	556	27800						
Sex								
Female	232 (41.7)	13,396 (48.2)	1.00	reference		1.00	reference	
Male	324 (58.3)	14,404 (51.8)	1.30	1.10–1.54	<.01	1.27	1.07–1.51	<.01
Race and ethnicity								
Non-Hispanic White	267 (48.0)	10,141 (36.5)	1.00	reference		1.00	reference	
Non-Hispanic Black	4 (0.7)	2,238 (8.1)	0.07	0.03–0.18	<.01	0.07	0.03–0.19	<.01
Hispanic	238 (42.8)	12,310 (44.3)	0.73	0.62–0.88	<.01	0.82	0.65–1.02	0.07
Non-Hispanic Asian	41 (7.4)	2,713 (9.8)	0.57	0.41–0.80	<.01	0.65	0.45–0.94	0.02
Other	6 (1.1)	398 (1.4)	0.57	0.25–1.29	0.18	0.62	0.27–1.40	0.25
Birth weight (grams)								
250–2499	25 (4.5)	1,652 (5.9)	0.79	0.52–1.20	0.27	0.80	0.49–1.28	0.35
2500–2999	69 (12.4)	4,289 (15.4)	0.84	0.64–1.11	0.21	0.88	0.66–1.16	0.36
3000–3499	202 (36.3)	10,538 (37.9)	1.00	reference		1.00	reference	
3500–3999	182 (32.7)	8,281 (29.8)	1.15	0.94–1.40	0.19	1.07	0.87–1.31	0.54
≥ 4000	78 (14.0)	3,040 (10.9)	1.34	1.03–1.74	0.03	1.19	0.91–1.56	0.21
Every 100 g					<.01	1.02	1.00–1.03	0.05
Every 500 g					<.01	1.09	1.00–1.18	0.05
Gestational age (weeks)								
22–36	42 (7.6)	2,485 (8.9)	0.83	0.60–1.15	0.26	0.96	0.67–1.37	0.81
37–41	421 (75.7)	20,733 (74.6)	1.00	reference		1.00	reference	
42–44	56 (10.1)	2,667 (9.6)	1.03	0.78–1.37	0.82	1.02	0.77–1.36	0.87
Unknown	37 (6.7)	1,915 (6.9)	0.95	0.68–1.34	0.77	0.98	0.70–1.38	0.91
Birth plurality								
Singleton	541 (97.3)	27,145 (97.6)	1.00	reference		1.00	reference	
Multiple	15 (2.7)	655 (2.4)	1.15	0.68–1.93	0.60	1.36	0.77–2.40	0.29
Birth order								
1st	211 (37.9)	11,186 (40.2)	1.00	reference		1.00	reference	
2nd	200 (36.0)	8,783 (31.6)	1.21	0.99–1.47	0.06	1.21	0.98–1.49	0.07
3rd and higher	145 (26.1)	7,831 (28.2)	0.98	0.79–1.22	0.86	1.01	0.79–1.29	0.94
Mode of delivery								
Vaginal	434 (78.1)	21,727 (78.2)	1.00	reference		1.00	reference	
Cesarean	122 (21.9)	6,073 (21.8)	1.01	0.82–1.23	0.96	1.03	0.81–1.30	0.81
Year of birth								
1978–1982	91 (16.4)	4,550 (16.4)	1.00	0.76–1.32	1.00	1.03	0.59–1.78	0.92
1983–1987	111 (20.0)	5,550 (20.0)	1.00	0.77–1.30	1.00	1.03	0.60–1.76	0.92
1988–1992	122 (21.9)	6,100 (21.9)	1.00	reference		1.00	reference	
1993–2014	232 (41.7)	11,600 (41.7)	1.00	0.80–1.25	1.00	1.00	0.80–1.26	1
Maternal age (years)								
< 20	56 (10.1)	3,194 (11.5)	0.85	0.63–1.15	0.29	0.94	0.67–1.32	0.74
20–24	134 (24.1)	7,385 (26.6)	0.88	0.70–1.11	0.27	0.91	0.72–1.16	0.44
25–29	166 (29.9)	8,040 (28.9)	1.00	reference		1.00	reference	
30–34	139 (25.0)	6,054 (21.8)	1.11	0.89–1.40	0.36	1.09	0.86–1.37	0.47
≥ 35	61 (11.0)	3,127 (11.2)	0.94	0.70–1.27	0.71	0.94	0.69–1.29	0.72
Maternal education (years)								
< 9	41 (7.4)	2,381 (8.6)	1.05	0.72–1.52	0.81	1.15	0.77–1.72	0.49
9–11	68 (12.2)	3,155 (11.3)	1.31	0.95–1.81	0.10	1.42	1.02–1.98	0.04
12	87 (15.6)	5,295 (19.0)	1.00	reference		1.00	reference	
13–15	81 (14.6)	3,384 (12.2)	1.46	1.07–1.98	0.02	1.40	1.03–1.91	0.03
≥ 16	72 (12.9)	3,185 (11.5)	1.38	1.00–1.89	0.05	1.19	0.85–1.66	0.32
Unknown	207 (37.2)	10,400 (37.4)	1.21	0.94–1.56	0.14	1.13	0.65–1.97	0.65
Continous (exclude unknown)				0.12	1.03	0.92–1.15	0.57	
Mother's place of birth								
United States	358 (64.4)	16,705 (60.1)	1.00	reference		1.00	reference	
Foreign	198 (35.6)	11,095 (39.9)	0.83	0.70–0.99	0.04	0.87	0.70–1.09	0.22
Maternal history of miscarriage/stillbirth								
No	459 (82.6)	23,027 (82.8)	1.00	reference		1.00	reference	
Yes	97 (17.4)	4,731 (17.0)	1.03	0.82–1.28	0.80	0.99	0.79–1.24	0.92
Unknown	0	42 (0.2)	NA	NA	0.97	NA	NA	0.97
Maternal complication during pregnancy								
No	455 (81.8)	23,069 (83.0)	1.00	reference		1.00	reference	
Yes	90 (16.2)	4,172 (15.0)	1.09	0.87–1.37	0.44	1.13	0.89–1.44	0.33
Unknown	11 (2.0)	559 (2.0)	1.00	0.55–1.83	0.99	1.08	0.24–4.84	0.92
Maternal history of cesarean delivery								
No	506 (91.0)	25,022 (90.0)	1.00	reference		1.00	reference	
Yes	41 (7.4)	2,310 (8.3)	0.88	0.64–1.21	0.43	0.79	0.54–1.14	0.21
Unknown	9 (1.6)	468 (1.7)	0.95	0.49–1.85	0.88	0.87	0.18–4.17	0.86

NA: Odds ratio was unevaluable due to the lack of cases with “unknown” maternal history of miscarriage or stillbirth

^a^The adjusted odds ratios were derived from multivariable logistic regression models in which all the variables listed in the table were included simultaneously

Apart from race and ethnicity, a heavier birthweight was significantly associated with an increased risk of Ewing sarcoma overall (OR = 1.09, 95% CI 1.00–1.18 for each 500 g increase in birthweight in the multivariable model), although non-significantly elevated risks were apparent for multiple births and second birth order compared to first (Table 2). Compared with individuals whose mothers received high school education, those born to mothers with 9–11 years or 13–15 years of education appeared to have a higher risk of Ewing sarcoma (Table 2).

All four Black Ewing sarcoma cases were classified as having bone tumor, and none had soft tissue sarcoma. Asian cases were also less likely to have soft tissue sarcomas when compared to non-Hispanic White subjects (OR = 0.27, 95% CI 0.10–0.71). Risks for both bone tumor and soft tissue sarcoma were lower among Hispanic subjects than in non-Hispanic White subjects, though not significantly (Supplementary Table 1 and 2). Birth order (second compared to first) was a risk factor for bone tumors only (Supplementary Table 1).

About one-third of the cases exhibited distant or metastatic disease apart from the primary tumor site at the time of diagnosis. Racial and ethnic differences appeared to be more profound for the risk of metastatic Ewing sarcoma, with non-Hispanic White individuals exhibiting a much higher risk of metastatic disease than individuals from the other racial and ethnic groups (Supplemental Tables 3, 4). When comparing non-Hispanic White subjects to all others as a reference group, White subjects had a higher risk of metastatic Ewing sarcoma (multivariable adjusted OR = 2.11 95% CI 1.44–3.09, not displayed in a table). The mothers of metastatic cases were more likely to be born outside the United States (OR = 1.53, 95% CI 1.03–2.25), while mothers of cases with localized or regional disease were less likely to be born outside the United States (OR = 0.71, 95% CI 0.54–0.94) (Supplemental Tables 3, 4).

Data for California families were available for 353 probands. Only a single family had multiple cancers – a young adult diagnosed with Ewing sarcoma had a younger sibling who was diagnosed with rhabdomyosarcoma as a child. Based on SEER age-specific cancer incidence rates calculated among observed family members (as previously described [5]), we would expect less than one case among siblings (0.557), yielding an SIR of 1.79 (95% CI 0.47, 6.85), providing no evidence of family-based cancer predisposition alleles in our study population. Data on other childhood sarcomas were mixed. There were no families with multiple cancers with a synovial sarcoma proband (out of 127 families). Four families with osteosarcoma proband (out of 575 families) had multiple cancers (SIR = 3.83, 95% CI 1.44–10.2), and 11 families with rhabdomyosarcoma proband (of 719 families) had multiple cancers (SIR = 6.47, 95% CI 3.01–13.95).

## Discussion

Given the rarity of Ewing sarcoma, epidemiologic evaluations of the etiology of this disease are limited, with the most notable and longstanding observation that the disease has a profoundly lower incidence among those of self-described African ancestry compared to other groups, particularly when compared to those of European ancestry [7]. Our study confirms and quantifies this at about a 14-fold risk difference. We also observed that Asian and Hispanic individuals also have a significantly decreased risk. While a lower incidence of Ewing sarcoma in Asia was noted as early as 1980 in China [8], our results quantify this risk difference within a single population (i.e., the birth cohort of California) as nearly *one-half*, although without further specification on countries of origin due to the limited sample size. Hispanic individuals have an intermediate risk, which may reflect a combination of risks from admixture of European, Amerindian, and African ancestral groups. These results are of great interest to genome-wide association studies which have identified strong genetic risk factors for Ewing sarcoma, particularly those in proximity to GGAA microsatellite repeats. The differencing structure of such repeats is thought to explain, at least in part, a decreased risk of Ewing sarcoma among those of African ancestry [9, 10]. We also found that the elevated risk among non-Hispanic White individuals was more extreme for patients exhibiting metastatic disease, which is a poor prognostic indicator and may suggest a biologically more aggressive disease. When examining localized or regional disease there were not any significant racial and ethnic differences apart from Black subjects. Patients whose mothers were born outside the United States were significantly more likely to present with metastatic disease and less likely to present with non-metastatic disease, which may reflect different access to healthcare resources and different care-seeking behavior [11]. Our family-based evaluation identified strong evidence for the involvement of familial predisposition alleles in the etiology of rhabdomyosarcoma and osteosarcoma, but no evidence for Ewing and synovial sarcomas. This result places more emphasis on the potential impact of low penetrance alleles, which were previously identified with relatively small sample sizes from genome-wide association studies [9, 10]. Further analyses are warranted to assess whether these ancestry variable genomic attributes account for the differences in risk between the range of racial and ethnic groups that make up the diverse population in the United States, or whether environmental factors play an important etiological role.

Besides race and ethnicity, we note a significantly increased risk of Ewing sarcoma in those with a heavier birthweight, which was not identified by prior analyses to our knowledge. In fact, a recent meta-analysis reported an insignificant finding combining results from four studies of Ewing bone tumor. [4] Another pooled analysis cited Ewing sarcoma as one of relatively few types of childhood cancer that are not associated with birthweight [12], based on 202 cases with limited overlap (those diagnosed at the age of ≤ 4 years) with our study population. The increased risk associated with high birthweight was similar in patients with bone and soft tissue tumors (Supplemental Tables 1, 2), suggesting a global or general effect regardless of tissue of origin.

Our study exhibits strengths and weaknesses. Strengths manifest through a large population base for the ascertainment of a rare cancer, a large number of controls selected from statewide birth records, and the extremely low likelihood for selection bias (no case or control had to be tracked or consented to participate) and information bias related to birth characteristics (birth records are routinely collected, prior to the development of cancer). As for weaknesses, we acknowledge the lack of information on other individual characteristics, such as diet and environmental chemical exposures. Our findings however are robust and should stimulate further investigations into the etiology of Ewing sarcoma.

## Supplementary Information

Below is the link to the electronic supplementary material.

Supplementary file1 (XLSX 46 KB)

## Data Availability

The collection of cancer incidence data used in this study was supported by the California Department of Public Health as part of the statewide cancer reporting program mandated by California Health and Safety Code Section 103885; the National Cancer Institute’s Surveillance, Epidemiology and End Results Program under contract HHSN261201000140C awarded to the Cancer Prevention Institute of California, contract HHSN261201000035C awarded to the University of Southern California, and contract HHSN261201000034C awarded to the Public Health Institute; and the Centers for Disease Control and Prevention’s National Program of Cancer Registries, under agreement U58DP003862-01 awarded to the California Department of Public Health. The California Department of Public Health is not responsible for the results or conclusions drawn by the authors of this publication. The primary data used in the analysis cannot be released publicly based on IRB-approved procedures established for the analysis.

## Abbreviations

CCR: California Cancer Registry–a statewide registry that includes information on the incidence of primary cancers for all California residents
ICD-O-3: International Classification of Diseases for Oncology, 3^rd^ edition
OR (95% CI): Odds ratio (95% confidence interval)
SEER: Surveillance, Epidemiology and End Results program, a national program to track cancer incidence and mortality in the United States
SIR: Standardized Incidence Ratio–the ratio between observed and expected cancer cases (expected are based on SEER rates)
